# Patient-Reported Outcomes in Phase 3 Clinical Trials for Blood Cancers: A Systematic Review

**DOI:** 10.1001/jamanetworkopen.2024.14425

**Published:** 2024-06-03

**Authors:** Kishan Patel, Alexandra Ivanov, Tajmah Jocelyn, Andrew Hantel, Jacqueline S. Garcia, Gregory A. Abel

**Affiliations:** 1 Department of Internal Medicine, Brigham & Women’s Hospital, Boston, Massachusetts; 2 Division of Population Sciences, Dana-Farber Cancer Institute, Boston, Massachusetts; 3 Center for Clinical Investigation, Brigham & Women’s Hospital, Boston, Massachusetts; 4 Division of Hematologic Malignancies, Dana-Farber Cancer Institute, Boston, Massachusetts

## Abstract

**Importance:**

Published research suggests that patient-reported outcomes (PROs) are neither commonly collected nor reported in randomized clinical trials (RCTs) for solid tumors. Little is known about these practices in RCTs for hematological malignant neoplasms.

**Objective:**

To evaluate the prevalence of PROs as prespecified end points in RCTs of hematological malignant neoplasms, and to assess reporting of PROs in associated trial publications.

**Evidence Review:**

All issues of 8 journals known for publishing high-impact RCTs (*NEJM, Lancet, Lancet Hematology, Lancet Oncology, Journal of Clinical Oncology, Blood, JAMA, and JAMA Oncology*) between January 1, 2018, and December 13, 2022, were searched for primary publications of therapeutic phase 3 trials for adults with hematological malignant neoplasms. Studies that evaluated pretransplant conditioning regimens, graft-vs-host disease treatment, or radiotherapy as experimental treatment were excluded. Data regarding trial characteristics and PROs were extracted from manuscripts and trial protocols. Univariable analyses assessed associations between trial characteristics and PRO collection or reporting.

**Findings:**

Ninety RCTs were eligible for analysis. PROs were an end point in 66 (73%) trials: in 1 trial (1%) as a primary end point, in 50 (56%) as a secondary end point, and in 15 (17%) as an exploratory end point. PRO data were reported in 26 of 66 primary publications (39%): outcomes were unchanged in 18 and improved in 8, with none reporting worse PROs with experimental treatment. Trials sponsored by for-profit entities were more likely to include PROs as an end point (49 of 55 [89%] vs 17 of 35 [49%]; *P* < .001) but were not significantly more likely to report PRO data (20 of 49 [41%] vs 6 of 17 [35%]; *P* = .69). Compared with trials involving lymphoma (18 of 29 [62%]) or leukemia or myelodysplastic syndrome (18 of 28 [64%]), those involving plasma cell disorders or multiple myeloma (27 of 30 [90%]) or myeloproliferative neoplasms (3 of 3 [100%]) were more likely to include PROs as an end point (*P* = .03). Similarly, compared with trials involving lymphoma (3 of 18 [17%]) or leukemia or myelodysplastic syndrome (5 of 18 [28%]), those involving plasma cell disorders or multiple myeloma (16 of 27 [59%]) or myeloproliferative neoplasms (2 of 3 [67%]) were more likely to report PROs in the primary publication (*P* = .01).

**Conclusions and Relevance:**

In this systematic review, almost 3 of every 4 therapeutic RCTs for blood cancers collected PRO data; however, only 1 RCT included PROs as a primary end point. Moreover, most did not report resulting PRO data in the primary publication and when reported, PROs were either better or unchanged, raising concern for publication bias. This analysis suggests a critical gap in dissemination of data on the lived experiences of patients enrolled in RCTs for hematological malignant neoplasms.

## Key Points

**Question:**

How frequently are patient-reported outcomes (PROs) used as end points in randomized clinical trials (RCTs) of hematological malignant neoplasms, and are these PROs reported in primary trial publications?

**Findings:**

In this systematic review of 90 therapeutic RCTs of hematological malignant neoplasms, 66 included PROs as an end point, but only 1 as a primary end point. PRO data were reported in 26 of the 66 publications: PROs were unchanged in 18 studies and improved in 8, with none reporting worse PROs with experimental treatment.

**Meaning:**

These results suggest there is widespread collection of PROs for blood cancer trials but limited reporting of PRO data in associated primary trial publications, raising concerns for publication bias.

## Teaser

This systematic review of randomized clinical trials investigating hematological malignant neoplasms examines how often patient-reported outcomes are included in results.

## Introduction

There is growing interest in incorporating patient-reported outcomes (PROs) into clinical trials as a way to better capture health-related quality of life (HRQOL) and other patient-centered end points beyond survival.^1,2^ PROs are the criterion standard for the assessment of subjective symptoms, as patients are in the best position to comment on their own lived experience of treatment. Indeed, several studies have shown that physicians routinely underestimate the incidence and severity of patients’ symptoms,^3,4,5,6,7,8,9^ and that PROs are more often concordant with overall health status than physician-led toxicity reports.^10^ Both the American Society of Clinical Oncology (ASCO) and the European Society of Medical Oncology (ESMO) include PRO data among the parameters considered for evaluation of clinical value of cancer-related drugs, with additional points conferred to drugs that can reliably demonstrate improvement in HRQOL and other PROs.^11,12^

The use of PROs as end points in randomized clinical trials (RCTs) is widely variable in oncology, and the collection and reporting of PRO data has been considered to be suboptimal.^13,14,15^ For example, a systematic review by Marandino and colleagues^14^ found that among phase 3 trials for solid tumors published between 2012 and 2016, HRQOL was not an end point in 47% of the trials, and related data were commonly underreported in the associated primary trial publications. Moreover, prior studies have suggested that even when clinical trials do collect PROs, they are infrequently assessed after disease progression and rarely followed until the end of a patient’s life.^16^

In contrast, there are sparse data about the use of PROs in RCTs for hematological malignant neoplasms. Given the significant morbidity associated with many blood cancers as well as the adoption of prolonged, non–fixed duration treatment in multiple hematological malignant neoplasms, PROs are critical to evaluate the effectiveness of drugs in this patient population. A prior study^17^ suggested that PROs were infrequently measured for pivotal blood cancer drugs that received FDA approval between January 2016 and May 2020. While an important contribution, a better denominator is all trials that were published in high-impact journals, given that not all trials lead to FDA approval. Moreover, key features of PRO data, such as the timing of data collection and associations of PRO collection and reporting with trial characteristics, have not been described. In this context, we hypothesized that PROs would be collected in a minority of therapeutic RCTs for blood cancers, and that only a subset of these trials would report PRO data in their primary publication.

## Methods

### Reporting, Information Sources, and Search Strategy

The present study followed the Preferred Reporting Items for Systematic Reviews and Meta-analyses (PRISMA) reporting guidelines. This study was exempt from institutional review board review because it involved the collection or study of existing, deidentified data.

The following 8 journals were selected by study team consensus for review because of their history of publishing high-impact blood cancer RCTs: *New England Journal of Medicine (NEJM), Lancet, Lancet Hematology, Lancet Oncology, Journal of Clinical Oncology, Blood, JAMA, and JAMA Oncology*. Journal issues published between January 1, 2018, and December 13, 2022, were manually searched for primary publications of therapeutic phase 3 trials for adults with hematological malignant neoplasms. For trials published in 2018 and 2019, secondary peer-reviewed publications reporting PROs were subsequently identified using an advanced PubMed search (run on March 22, 2023) with the terms *(trial name)* AND *((disease)* OR *(drug))* AND *((quality of life)* OR *(patient reported outcome)* OR *(PRO)* OR *(QOL)* OR *(HRQOL))*, and secondary abstracts reporting PROs at the American Society of Hematology (ASH) and European Hematology Association (EHA) annual meetings were identified using a search (run on March 7, 2024) with the terms *(trial name)* AND *((quality of life)* OR *(PRO))* in the respective abstract libraries. The 2018-2019 years were selected to maximize the probability of a secondary publication specific to PROs. The quality of the data sources was based on the Oxford Center for Evidence-Based Medicine’s Levels of Evidence and Grades of Recommendation; since all the included studies were phase 3 RCTs, all had a rating of 1.^18^

### Eligibility Criteria

Eligible trials were therapeutic, phase 3 RCTs that included adult patients with acute myeloid leukemia (AML), acute promyelocytic leukemia (APL), acute lymphoblastic leukemia (ALL), chronic lymphocytic leukemia (CLL), chronic myeloid leukemia (CML), chronic myelomonocytic leukemia (CMML), hairy cell leukemia, Hodgkin lymphoma, non-Hodgkin lymphoma, multiple myeloma, Waldenstrom macroglobulinemia, light-chain amyloidosis, myelodysplastic syndromes (MDS), and myeloproliferative neoplasms (MPNs) including polycythemia vera, essential thrombocythemia, and primary myelofibrosis. Studies that evaluated immunosuppressive regimens for graft-vs-host disease (GVHD), conditioning regimens prior to hematopoietic stem cell transplantation, or radiotherapy as experimental treatment were excluded. Studies for which the original trial protocol document was not available were also excluded.

### Data Extraction

Two investigators (K.P. and A.I.) independently extracted data from each eligible clinical trial protocol and publication; discrepancies were resolved by consensus arbitration with a third investigator (G.A.). Concordance was assessed by calculation of κ statistics for major extracted variables. For each study, extracted data included: journal of publication, date of publication, type of malignant neoplasm, disease stage (first-line treatment, relapsed or refractory treatment, or maintenance treatment after remission or transplantation), number of study arms, single or multinational study, primary sponsor (pharmaceutical company, hospital or academic institution, or trial or cooperative group, as listed on ClinicalTrials.gov), type of experimental treatment (chemotherapy, targeted therapy or immunotherapy, cellular therapy, growth factor or erythroid maturation agent, or transplantation), duration of therapy (time-limited or indefinite), masking (double-masked or open label), primary end point, and study design (superiority or noninferiority). Studies were labeled as positive (when 1 or more primary end points were met) or negative. Trials were considered for-profit when sponsored by a pharmaceutical company and as nonprofit when sponsored by a hospital, academic institution, or cooperative group.

Information regarding PROs was collected from the primary trial publication as well as the trial protocol. The publication through which the trial was included in the analysis was considered the primary trial publication. Extracted information included: incorporation of PROs as an end point (primary, secondary, exploratory); the instruments used to collect PRO data (eg, European Organization for Research and Treatment Quality of Life of Cancer Patients [EORTC-QLQ-C30], EuroQol Research Foundation 5 Dimensions [EQ-5D], Functional Assessment of Cancer Therapy-General [FACT-G]); the timing of PRO measurement (ie, during treatment, at the end-of-treatment visit, and/or after the end-of-treatment visit); whether the PRO data were reported in the primary trial publication or data supplement; and the reported effect of the experimental treatment on PROs (ie, improved, no change, or worse). For the timing of PRO measurement, trials were characterized as having collected PRO data after the end-of-treatment visit only if PRO data were collected more than 1 month afterward, with the intention to screen out trials where the only additional PRO datapoint was at a single safety follow-up visit within one month of the end-of-treatment.

Secondary publications and ASH or EHA abstracts reporting PROs were identified for clinical trials published in 2018 and 2019 where PROs were included as an end point but PRO data had not been reported in the primary publication. These years were selected to allow for sufficient time for a secondary analysis dedicated to PROs to be published. Delays in publication time between primary and secondary publications were calculated in months.

### Statistical Analysis

Associations between trial characteristics and the collection and reporting of PROs in the primary publication were assessed using χ^2^ or Fisher exact test. Significance was considered at *P* < .05 and analyses were 2-tailed; there was no adjustment for multiple comparisons. Statistical analysis was performed using Stata version 17 (StataCorp).

## Results

### Study Selection and Concordance

Ninety eligible phase 3 clinical trials were identified from the 8 journals (Figure).^19,20,21,22,23,24,25,26,27,28,29,30,31,32,33,34,35,36,37,38,39,40,41,42,43,44,45,46,47,48,49,50,51,52,53,54,55,56,57,58,59,60,61,62,63,64,65,66,67,68,69,70,71,72,73,74,75,76,77,78,79,80,81,82,83,84,85,86,87,88,89,90,91,92,93,94,95,96,97,98,99,100,101,102,103,104,105,106,107,108^ Concordance κ for investigator assessments of whether PROs were a prespecified end point, whether PROs were reported in the primary trial publication, and reported change in the PRO with the experimental treatment were 0.90, 0.96, and 0.97, respectively.

**Figure.**
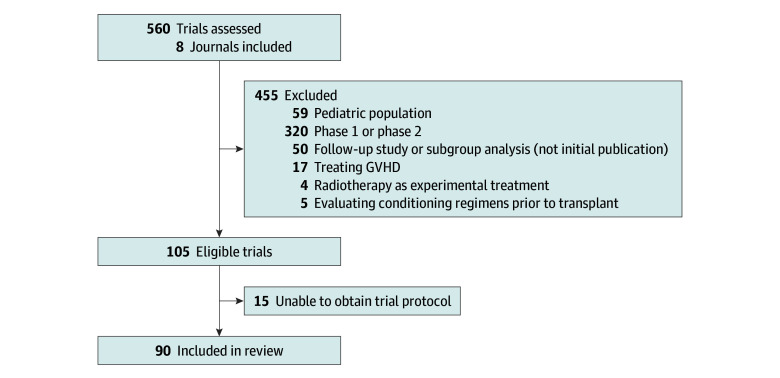
PRISMA Flowchart PRISMA flowchart summarizing review and selection process, including the number of excluded trials and the rationale for trial exclusion. GVHD indicates graft-vs-host disease.

### Study Characteristics

Study characteristics are provided in Table 1, and individual studies analyzed are listed in eTable 1 in Supplement 1. The journals with the highest number of primary trial publications were the *Journal of Clinical Oncology* (25 trials [28%]) and the *NEJM* (20 trials [22%]). Overall, 30 (33%) reported data for plasma cell dyscrasias or multiple myeloma, 29 (32%) lymphoma, 28 (31%) leukemia or MDS, and 3 (3%) MPNs. Fifty-five trials (61%) were sponsored by a for-profit entity, while 35 (39%) were sponsored by a nonprofit entity. Eighty-three (92%) were superiority studies, while 7 (8%) were noninferiority studies. Seventy-one trials (79%) were open label, and most studies (77 [86%]) involved multiple nations. The most common therapeutic stage was first-line (49 [54%]). Experimental treatments included chemotherapy (22 [24%]), targeted therapy or immunotherapy (60 [67%]), cellular therapy (3 [3%]), growth factor or erythroid maturation agents (2 [2%]), transplantation (1 [1%]), or a combination (2 [2%]). Most trials (65 [72%]) used progression-free survival (PFS), event-free survival (EFS), or disease-free survival (DFS) as their primary end points. Sixty-two (69%) of the studies were positive, meeting 1 or more of their primary end points.

**Table 1.** Characteristics of 90 Primary Publications Included in the Analysis

Characteristic	Studies, No. (%)
Year of primary manuscript	
2018	18 (20)
2019	24 (27)
2020	18 (20)
2021	15 (17)
2022	15 (17)
Primary manuscript journal	
* NEJM*	20 (22)
* Lancet*	8 (9)
* Lancet Oncology*	17 (19)
* Lancet Hematology*	11 (12)
* Journal of Clinical Oncology*	25 (28)
* Blood*	8 (9)
* JAMA*	0
* JAMA Oncology*	1 (1)
Sources of funding	
Profit	55 (61)
Nonprofit	35 (39)
Type of malignancy	
Plasma cell dyscrasia	30 (33)
Multiple myeloma	25 (28)
Smoldering multiple myeloma	1 (1)
Waldenstrom macroglobulinemia	2 (2)
Light-chain amyloidosis	2 (2)
Lymphoma	29 (32)
Diffuse large B-cell lymphoma	9 (10)
Mantle cell lymphoma	2 (2)
Follicular lymphoma	4 (4)
Hodgkin lymphoma	3 (3)
T-cell lymphoma	4 (4)
Primary CNS lymphoma	1 (1)
Multiple	6 (7)
Leukemia	28 (31)
Acute myeloid leukemia	9 (10)
Acute promyelocytic leukemia	1 (1)
Acute lymphoblastic leukemia	2 (2)
Chronic lymphocytic leukemia	10 (11)
Chronic myeloid leukemia	2 (2)
Myelodysplastic syndrome	3 (3)
Multiple	1 (1)
Myeloproliferative neoplasm	3 (3)
Essential thrombocythemia	1 (1)
Myelofibrosis	1 (1)
Multiple	1 (1)
Study design	
Superiority	83 (92)
Noninferiority	7 (8)
Masking	
Open label	71 (79)
Double-masked	19 (21)
Nations included	
Single nation	13 (14)
China	2 (2)
France	1 (1)
Germany	2 (2)
Italy	1 (1)
United Kingdom	1 (1)
US	6 (7)
Multiple nations	77 (86)
Type of experimental therapy	
Chemotherapy	22 (24)
Targeted therapy or Immunotherapy	60 (67)
Cellular therapy	3 (3)
Growth factor or erythroid maturation agent	2 (2)
Transplant	1 (1)
Multiple	2 (2)
Disease stage	
First-line	49 (54)
Maintenance after first-line	4 (4)
Maintenance after transplant	3 (3)
Relapsed/refractory	29 (32)
First-line and maintenance	1 (1)
First-line and relapsed/refractory	4 (4)
Primary end point^a^	
PFS, EFS, or DFS	65 (72)
Overall survival	11 (12)
Response rate^b^	15 (17)
Relapse rate	1 (1)
Transfusion independence	2 (2)
MRD negativity	1 (1)
Maximum trough concentration	1 (1)
Time to thrombosis, hemorrhage, or death from vascular causes	1 (1)
Patient-reported outcomes	1 (1)
Spleen volume reduction	1 (1)
Positive results	
Yes^c^	62 (69)
No	28 (31)

Abbreviations: CNS, central nervous system; DFS, disease-free survival; EFS, event-free survival; MRD, minimal residual disease; *NEJM*, *New England Journal of Medicine*; PFS, progression-free survival.

^a^ Categories not mutually exclusive.

^b^ Includes any type of response rate, including complete response, overall response rate, very good partial response, major erythroid response, hematological response, kidney response, major molecular response.

^c^ If study had multiple primary end points, it was considered positive if the experimental treatment demonstrated statistically significant improvement in at least one primary end point.

### Inclusion of PROs as End Points

Twenty-four clinical trials (27%) did not include PROs as a prespecified end point. Among the 66 (73%) that did, only 1 RCT (1%) included PROs as a primary end point, 50 (56%) had PROs as a secondary end point, and 15 (17%) as an exploratory end point (Table 2). Trials that were sponsored by for-profit entities were more likely to include PROs as an end point compared with trials sponsored by nonprofit entities (49 of 55 [89%] vs 17 of 35 [49%]; *P* < .001) (Table 3). Trials involving plasma cell dyscrasias and/or multiple myeloma (27 of 30 [90%]) or MPNs (3 of 3 [100%]) were more likely to include PROs as an end point compared with trials involving lymphoma (18 of 29 [62%]) or leukemia and/or MDS (18 of 28 [64%]) (*P* = .03). There was no association between disease stage or positive primary end point results and inclusion of PROs as an end point. Lastly, trials involving time-limited treatment were less likely to include PROs as an end point compared with trials with indefinite treatment (24 of 43 [56%] vs 42 of 47 [89%]; *P* < .001).

**Table 2.** PROs in Phase 3 Studies of Hematologic Malignant Neoplasms

Characteristic	Studies, No. (%) (N = 90)
PRO listed as end point	66 (73)
Primary	1 (1)
Secondary	50 (56)
Exploratory	15 (17)
PRO not an end point	24 (27)
**Studies with PROs as end points (n = 66)**	
Type documented^a^	
General HRQOL	65 (98)
Disease-specific metric	36 (55)
Symptom-specific metric	21 (32)
Collected during treatment	
Yes	64 (97)
No	1 (2)
Not recorded in protocol	1 (2)
Collected at end-of-treatment visit	
Yes	61 (92)
No	3 (5)
Not recorded in protocol	2 (3)
Collected after end-of-treatment visit, during follow-up	
Yes	39 (59)
No	24 (36)
Not recorded in protocol	3 (5)
Reported in the primary publication (including data supplement)	
Yes	26 (39)
No	40 (61)
**Subcohorts of studies with PROs as end point**	
2018-2019 studies with PRO data not reported in primary publication (n = 19)	
PRO data reported in a secondary publication or ASH/EHA meeting	
Yes	8 (42)
No	11 (58)
Studies with PROs reported in primary publication, secondary publication, or ASH/EHA meeting (n = 34)	
Change in PROs with experimental treatment	
Improvement	13 (38)
No change	21 (62)
Worsening	0

Abbreviations: ASH, American Society of Hematology; EHA, European Hematology Association; HRQOL, health-related quality of life; PRO, patient-reported outcomes.

^a^ See Methods sections for list of measures.

**Table 3.** Associations Between Trial Characteristics and Inclusion of PROs as an End Point

Characteristic	Studies, No. (%)^a^		*P* value
	PRO end point	No PRO end point	
Source of funding			
Profit	49 (89)	6 (11)	<.001
Nonprofit	17 (49)	18 (51)	
Type of malignant neoplasm			
Plasma cell dyscrasia	27 (90)	3 (10)	.03
Lymphoma	18 (62)	11 (38)	
Leukemia	18 (64)	10 (36)	
MPN	3 (100)	0	
Disease stage^b^			
First-line	33 (67)	16 (33)	.23
Relapsed or refractory	24 (83)	5 (17)	
Maintenance	4 (57)	3 (43)	
Positive study			
Yes	46 (74)	16 (26)	.78
No	20 (71)	8 (29)	
Time-limited treatment			
Yes	24 (56)	19 (44)	<.001
No	42 (89)	5 (11)	

Abbreviations: MPN, myeloproliferative neoplasms; PRO, patient-reported outcomes.

^a^ These percentages reflect in-row relative frequencies.

^b^ Studies which spanned across different disease stages (n = 5) were omitted from univariable analysis.

### Characteristics of PROs Included in Phase 3 Blood Cancer Trials

Of the 66 trials that included PROs as an end point, 65 (98%) collected general quality-of-life measures, including EORTC-QLQ-C30,^109^ EQ-5D,^110^ FACT-G,^111^ 36-Item Short Form Survey (SF-36),^112^ Patient Report Outcome Measurement Information System (PROMIS) Cancer SF7a,^113^ MD Anderson Symptom Inventory (MDASI),^114^ General Health Questionnaire (GHQ-12),^115^ Cancer Therapy Satisfaction Questionnaire (CTSQ),^116^ Treatment Satisfaction Questionnaire for Medication (TSQM),^116^ Patient Global Impressions (PGI-C),^117^ and Work Productivity and Impairment Questionnaire (WPAI).^118^ Thirty-six (55%) collected disease-specific measures, including EORTC-QLQ subscales (eg, myeloma module [MY20], CLL module [CLL16]),^109^ FACT subscales (eg, leukemia, lymphoma, multiple myeloma, bone marrow transplant),^111^ or MDASI subscales (eg, CLL, CML, etc).^114^ Twenty-one (32%) collected symptom-specific metrics, including EORTC-QLQ subscales (eg, Chemotherapy-Induced Peripheral Neuropathy [CIPN20], Elderly Cancer Patients [ELD14]),^109^ FACT subscales (eg, neurotoxicity [NTX], fatigue, pain, dyspnea, anemia),^111^ PROMIS subscales (fatigue),^113^ Brief Pain Inventory,^119^ and ItchyQoL.^120^ Details regarding the PROs utilized for each disease type are included in eTable 2 in Supplement 1. With respect to timing of PRO collection, 64 trials (97%) collected PRO data during treatment, 61 (92%) at the end-of-treatment visit, and 39 (59%) greater than 1 month after the end-of-treatment visit.

### Reporting of PRO Data in Primary and Secondary Publications

Of the 66 trials that included PROs as an end point, 26 (39%) reported these data in the primary trial publication. Of these, 8 (31%) reported improvement in PROs with the experimental treatment, while 18 (69%) reported no change. No trials reported worsening of PROs in the experimental treatment arm. Trials involving plasma cell dyscrasias and/or multiple myeloma (16 of 27 [59%]) or MPNs (2 of 3 [67%]) were more likely to report PRO data in the primary publication compared with trials involving lymphoma (3 of 18 [17%]) or leukemia and/or MDS (5 of 18 [28%]) (*P* = .01) (Table 4). There was no association between trial sponsorship, disease stage, or positive primary end point result with PRO reporting in primary publications. Furthermore, there was no association between positive primary end point result and whether improvement was reported in measured PROs. Trials involving time-limited treatment were less likely to report PRO results compared with trials involving indefinite treatment (4 of 24 [17%] vs 22 of 42 [52%]; *P* = .004).

**Table 4.** Associations Between Trial Characteristics and Reporting of PROs in Primary Publications (Among Studies That Included PROs as an End Point)

Characteristics	Studies, No. (%)^a^		*P* value
	PRO reported	PRO not reported	
Source of funding			
Profit	20 (41)	29 (59)	.69
Nonprofit	6 (35)	11 (65)	
Type of malignant neoplasm			
Plasma cell dyscrasia	16 (59)	11 (41)	.01
Lymphoma	3 (17)	15 (83)	
Leukemia	5 (28)	13 (72)	
MPN	2 (67)	1 (33)	
Disease stage^b^			
First-line	11 (33)	22 (67)	.22
Relapsed or refractory	7 (29)	17 (71)	
Maintenance	3 (75)	1 (25)	
Positive study			
Yes	19 (41)	27 (59)	.63
No	7 (35)	13 (65)	
Time-limited treatment			
Yes	4 (17)	20 (83)	.004
No	22 (52)	20 (48)	

Abbreviations: MPN, myeloproliferative neoplasms; PRO, patient-reported outcomes.

^a^ These percentages reflect in-row relative frequencies.

^b^ Studies which spanned across different disease stages (n = 5) were omitted from univariable analysis.

There were 19 trials published in 2018 and 2019 that included PROs as an end point but did not report PRO data in the primary trial publication. Of these, 6 (32%) had a secondary peer-reviewed publication with PROs by March 2023; the mean delay in publication time was 21 months. Four of these 6 trials (66%) reported improvement in PROs with the experimental treatment, while 2 (33%) reported no change and none reported worsening PROs with treatment. Of the 13 trials (68%) that did not have a secondary publication, only 2 reported comparative PRO data in an abstract published at a subsequent ASH or EHA annual meeting; of these, 1 (50%) reported improvement in PROs with experimental treatment, and 1 (50%) reported no change.

## Discussion

PROs are critical to understanding patients’ perspectives about the functional, psychological, and physical effects of treatment. In contrast to our hypothesis (and to a prior analysis with a different definition of high-impact protocols), we found that PROs were included as an end point in a majority of therapeutic RCTs for hematological malignant neoplasms. However, PRO results were rarely reported in the primary trial publication, and when reported, were either improved or unchanged with the experimental treatment. These results reveal a critical gap in the dissemination of data on the lived experiences of patients with hematological malignant neoplasms during high-impact phase 3 clinical trials.

We identified significant differences between blood cancer disease groups regarding the collection and reporting of PRO data. For example, RCTs involving plasma cell disorders or multiple myeloma were significantly more likely to both collect and report PRO data in the associated primary publication. This contrasted with RCTs of lymphoma, leukemia, and MDS, where slightly over half of trials included PROs as an end point, and only a minority reported PRO results in the primary trial publication. One possible explanation is the increased adoption of triplet and/or quadruplet regimens as standard-of-care for the treatment of multiple myeloma; many of these regimens are not time-limited and therefore involve prolonged drug exposure, which may incentivize a more nuanced understanding by researchers of patient-reported adverse effects over time. In contrast, there may be less interest in measuring PROs for patients with lymphoma and aggressive leukemia, where treatments are most often of fixed-duration and time-limited decrements in HRQOL can be seen as the price to pay for a potential cure.

We did not identify a significant difference in the collection or reporting of PROs based on the line of treatment. One could argue that PRO data are particularly relevant for patients with relapsed or refractory disease, where clinicians must carefully balance disease control with patient symptoms and drug-related toxic effects. Reassuringly, we found that most RCTs in the relapsed and refractory settings included a PRO measure as an end point; however, only a relatively small fraction of these reported PRO data in the primary trial publication. Given that in many cases, later lines of therapy are associated with only incremental benefits in overall survival or surrogate end points,^121^ these data argue that there should be a more concerted effort to report PRO data in RCTs of relapsed and refractory patients, to allow clinicians and patients to make better decisions about the potential effectiveness of continued treatment.

We found that almost all RCTs that included a PRO as an end point collected PRO data through the end-of-treatment visit. However, less than two-thirds of these collected any PRO data more than 1 month after the end-of-treatment visit, suggesting a limited understanding of how treatments may affect long-term HRQOL. This finding is consistent with a prior report that found that 76% of primarily solid tumor oncology clinical trials published in 3 major journals between 2015 and 2018 assessed QoL during treatment, but fewer than 25% of RCTs continued PRO data collection until disease progression and fewer than 3% until patient death.^16^ While longer-term PRO data collection is challenging and resource intensive, it can leverage the RCT infrastructure to provide valuable insight into the natural history of HRQOL for each disease, even after the conclusion of treatment.

Only 1 RCT in our analysis included PROs as a primary end point; the most popular primary end points were surrogate survival metrics (PFS, EFS, DFS), overall survival, and response rate. We did not identify any association between positive trial results and inclusion of PROs as an end point or reporting of PRO data in the primary publication. Notably, 26% of positive RCTs did not include a PRO among the study end points, and 59% of positive RCTs that did have a PRO end point did not report PRO data in the primary trial publication. These data suggest that PROs are not readily available for many treatment regimens that are subsequently incorporated into routine clinical practice. At the very least, investigators for RCTs of hematological malignant neoplasms should be encouraged to report these results if they are available at the time of trial publication.

Indeed, lack of supporting PRO data has been previously identified. For example, Davis and colleagues^122^ showed that between 2009 and 2013, only 10% of EMA cancer drug approvals had evidence of improved HRQOL for patients. This issue is further compounded by potential publication bias: in our analysis, among the studies that reported PRO data either in the primary trial publication or a subsequent manuscript or abstract, none reported worsening of PROs with the experimental treatment. Combined with prior studies suggesting that RCTs that fail to show improvement in PROs often frame PRO outcomes in an undeservedly favorable light,^123,124^ this finding suggests a need for more stringent collecting and reporting requirements for PROs in blood cancer RCTs, such that detrimental impacts on HRQOL cannot be easily omitted from trial publications.

### Limitations

Our analysis has limitations. First, our data were limited to a subset of journals and calendar years, and our sample of RCTs is likely not fully representative of all therapeutic phase 3 trials for hematological malignant neoplasms. Our choice to focus on a subset of high-impact journals was intended to isolate RCTs that were most likely to inform clinical practice; however, we recognize that our study population is therefore likely to be enriched for RCTs that were able to meet their primary end points or demonstrate other hard outcomes. Second, given the relatively short timeframe included in our study, we were unable to assess whether there have been changes in the collection and reporting of PROs over time, particularly after the publication of guideline statements for PROs for RCTs.^125,126^ Third, our statistical analysis was limited by relatively small sample size, and we were not able to adjust for multiple comparisons or conduct multivariable analyses. In particular, our univariable analyses for RCTs dedicated to MPNs were limited, as only 3 such trials were included in our systematic review. Furthermore, the small sample size precluded our ability to analyze associations between individual PROs and trial characteristics. Finally, our study did not include pediatric RCTs; given similar concerns about suboptimal utilization of PROs in pediatric trials,^127,128^ we believe that a secondary review focused on this population is warranted.

## Conclusions

In contrast to prior work, we found that almost 3 of every 4 therapeutic RCTs for hematological malignant neoplasms collected PRO data; however, only 1 RCT included PROs as a primary end point. Moreover, a minority of studies reported these data in the associated primary publication, and when reported, PROs were either better or unchanged, raising concerns for publication bias. Our analysis suggests a critical gap in dissemination of data on the lived experiences of patients with hematological malignant neoplasms during phase 3 trials, which may be addressed with more inclusive PRO design and more stringent reporting requirements.

## Notes

Supplement 1. **eTable 1.** List of Reviewed Trials **eTable 2.** Aggregated PROs Used in Reviewed RCTs

Supplement 2.
Data Sharing Statement
