# Supplementary material for: Patient-Reported Outcomes in Phase 3 Clinical Trials for Blood Cancers: A Systematic Review
Source: JAMA Netw Open. 2024 Jun 3;7(6):e2414425. doi: 10.1001/jamanetworkopen.2024.14425 (PMC11148691; doi:10.1001/jamanetworkopen.2024.14425)
Supplement: Supplement 1. — eTable 1. List of Reviewed Trials eTable 2. Aggregated PROs Used in Reviewed RCTs [file jamanetwopen-e2414425-s001.pdf]

Supplemental Online Content

Patel K, Ivanov A, Jocelyn T, Hantel A, Garcia JS, Abel GA. Patient reported outcomes in phase III clinical trials for blood cancers. *JAMA Netw Open*. 2024;7(6):e2414425. doi:10.1001/jamanetworkopen.2024.14425

eTable 1. List of Reviewed Trials

eTable 2. Aggregated PROs Used in Reviewed RCTs

This supplemental material has been provided by the authors to give readers additional information about their work.

© 2024 Patel K et al. *JAMA Network Open.*

Supplemental Table 1: List of Reviewed Trials
Organized by Disease Group

Title of Study	Year	Journal	NCT#	Malignancy (General)	Malignancy (Specific)
Hydroxycarbamide Plus Aspirin Versus Aspirin Alone in Patients With Essential Thrombocythemia Age 40 to 59 Years Without High-Risk Features	2018	Journal of Clinical Oncology	NCT00175838	MPN	Essential thrombocythemia
A randomized phase 3 trial of interferon-α vs hydroxyurea in polycythemia vera and essential thrombocythemia	2022	Blood	NCT01259856	MPN	Essential thrombocythemia, Polycythemia vera
Pacritinib vs Best Available Therapy, Including Ruxolitinib, in Patients With Myelofibrosis	2018	JAMA Oncology	NCT02055781	MPN	Myelofibrosis
Daratumumab-Based Treatment for Immunoglobulin Light-Chain Amyloidosis	2021	NEJM	NCT03201965	Plasma cell dyscrasia	Light-chain amyloidosis
Bortezomib, Melphalan, and Dexamethasone for Light-Chain Amyloidosis	2020	Journal of Clinical Oncology	NCT01277016	Plasma cell dyscrasia	Light-chain amyloidosis
Triplet Therapy, Transplantation, and Maintenance until Progression in Myeloma	2022	NEJM	NCT01208662	Plasma cell dyscrasia	Multiple myeloma
Daratumumab plus Lenalidomide and Dexamethasone for Untreated Myeloma	2019	NEJM	NCT02252172	Plasma cell dyscrasia	Multiple myeloma
Daratumumab plus Bortezomib, Melphalan, and Prednisone for Untreated Myeloma	2018	NEJM	NCT02195479	Plasma cell dyscrasia	Multiple myeloma
Isatuximab, carfilzomib, and dexamethasone in relapsed multiple myeloma (IKEMA): a multicentre, open-label, randomised phase 3 trial	2021	The Lancet	NCT03275285	Plasma cell dyscrasia	Multiple myeloma
Once-per-week selinexor, bortezomib, and dexamethasone versus twice-per-week bortezomib and dexamethasone in patients with multiple myeloma (BOSTON): a randomised, open-label, phase 3 trial	2020	The Lancet	NCT03110562	Plasma cell dyscrasia	Multiple myeloma
Carfilzomib, dexamethasone, and daratumumab versus carfilzomib and dexamethasone for patients with relapsed or refractory multiple myeloma (CANDOR): results from a randomised, multicentre, open-label, phase 3 study	2020	The Lancet	NCT03158688	Plasma cell dyscrasia	Multiple myeloma
Isatuximab plus pomalidomide and low-dose dexamethasone versus pomalidomide and low-dose dexamethasone in patients with relapsed and refractory multiple myeloma (ICARIA-MM): a randomised, multicentre, open-label, phase 3 study	2019	The Lancet	NCT02990338	Plasma cell dyscrasia	Multiple myeloma
Oral ixazomib maintenance following autologous stem cell transplantation (TOURMALINE-MM3): a double-blind, randomised, placebo-controlled phase 3 trial	2019	The Lancet	NCT02181413	Plasma cell dyscrasia	Multiple myeloma
Daratumumab plus pomalidomide and dexamethasone versus pomalidomide and dexamethasone alone in previously treated multiple myeloma (APOLLO): an open-label, randomised, phase 3 trial	2021	The Lancet Oncology	NCT03180736	Plasma cell dyscrasia	Multiple myeloma
Venetoclax or placebo in combination with bortezomib and dexamethasone in patients with relapsed or refractory multiple myeloma (BELLINI): a randomised, double-blind, multicentre, phase 3 trial	2020	The Lancet Oncology	NCT02755597	Plasma cell dyscrasia	Multiple myeloma
Carfilzomib or bortezomib in combination with lenalidomide and dexamethasone for patients with newly diagnosed multiple myeloma without intention for immediate autologous stem-cell transplantation (ENDURANCE): a multicentre, open-label, phase 3, randomised, controlled trial	2020	The Lancet Oncology	NCT01863550	Plasma cell dyscrasia	Multiple myeloma
Pomalidomide, bortezomib, and dexamethasone for patients with relapsed or refractory multiple myeloma previously treated with lenalidomide (OPTIMISMM): a randomised, open-label, phase 3 trial	2019	The Lancet Oncology	NCT01734928	Plasma cell dyscrasia	Multiple myeloma

© 2024 Patel K et al. *JAMA Network Open.*

Lenalidomide maintenance versus observation for patients with newly diagnosed multiple myeloma (Myeloma XI): a multicentre, open-label, randomised, phase 3 trial	2019	The Lancet Oncology	NCT01554852	Plasma cell dyscrasia	Multiple myeloma
Once weekly versus twice weekly carfilzomib dosing in patients with relapsed and refractory multiple myeloma (A.R.R.O.W.): interim analysis results of a randomised, phase 3 study	2018	The Lancet Oncology	NCT02412878	Plasma cell dyscrasia	Multiple myeloma
Addition of isatuximab to lenalidomide, bortezomib, and dexamethasone as induction therapy for newly diagnosed, transplantation-eligible patients with multiple myeloma (GMMG-HD7): part 1 of an open-label, multicentre, randomised, active-controlled, phase 3 trial	2022	The Lancet Hematology	NCT03617731	Plasma cell dyscrasia	Multiple myeloma
Addition of elotuzumab to lenalidomide and dexamethasone for patients with newly diagnosed, transplantation ineligible multiple myeloma (ELOQUENT-1): an open-label, multicentre, randomised, phase 3 trial	2022	The Lancet Hematology	NCT01335399	Plasma cell dyscrasia	Multiple myeloma
Melflufen or pomalidomide plus dexamethasone for patients with multiple myeloma refractory to lenalidomide (OCEAN): a randomised, head-to-head, open-label, phase 3 study	2022	The Lancet Hematology	NCT03151811	Plasma cell dyscrasia	Multiple myeloma
Subcutaneous versus intravenous daratumumab in patients with relapsed or refractory multiple myeloma (COLUMBA): a multicentre, open-label, non-inferiority, randomised, phase 3 trial	2020	The Lancet Hematology	NCT03277105	Plasma cell dyscrasia	Multiple myeloma
Response-adapted intensification with cyclophosphamide, bortezomib, and dexamethasone versus no intensification in patients with newly diagnosed multiple myeloma (Myeloma XI): a multicentre, open-label, randomised, phase 3 trial	2019	The Lancet Hematology	NCT01554852	Plasma cell dyscrasia	Multiple myeloma
Pembrolizumab plus lenalidomide and dexamethasone for patients with treatment-naive multiple myeloma (KEYNOTE-185): a randomised, open-label, phase 3 trial	2019	The Lancet Hematology	NCT02579863	Plasma cell dyscrasia	Multiple myeloma
Pembrolizumab plus pomalidomide and dexamethasone for patients with relapsed or refractory multiple myeloma (KEYNOTE-183): a randomised, open-label, phase 3 trial	2019	The Lancet Hematology	NCT02576977	Plasma cell dyscrasia	Multiple myeloma
Ixazomib as Postinduction Maintenance for Patients With Newly Diagnosed Multiple Myeloma Not Undergoing Autologous Stem Cell Transplantation: The Phase III TOURMALINE-MM4 Trial	2020	Journal of Clinical Oncology	NCT02312258	Plasma cell dyscrasia	Multiple myeloma
Randomized Trial Comparing Double Versus Triple Bortezomib-Based Regimen in Patients With Multiple Myeloma and Acute Kidney Injury Due to Cast Nephropathy	2020	Journal of Clinical Oncology	NCT01208818	Plasma cell dyscrasia	Multiple myeloma
Autologous Transplantation, Consolidation, and Maintenance Therapy in Multiple Myeloma: Results of the BMT CTN 0702 Trial	2019	Journal of Clinical Oncology	NCT01109004	Plasma cell dyscrasia	Multiple myeloma
Oral ixazomib, lenalidomide, and dexamethasone for transplant-ineligible patients with newly diagnosed multiple myeloma	2021	Blood	NCT01850524	Plasma cell dyscrasia	Multiple myeloma
Randomized Trial of Lenalidomide Versus Observation in Smoldering Multiple Myeloma	2020	Journal of Clinical Oncology	NCT01169337	Plasma cell dyscrasia	Smoldering multiple myeloma
Phase 3 Trial of Ibrutinib plus Rituximab in Waldenström's Macroglobulinemia	2018	NEJM	NCT02165397	Plasma cell dyscrasia	Waldenstrom macroglobulinemia
A randomized phase 3 trial of zanubrutinib vs ibrutinib in symptomatic Waldenström macroglobulinemia: the ASPEN study	2020	Blood	NCT03053440	Plasma cell dyscrasia	Waldenstrom macroglobulinemia
Polatuzumab Vedotin in Previously Untreated Diffuse Large B-Cell Lymphoma	2022	NEJM	NCT03274492	Lymphoma	DLBCL
Lisocabtagene maraleucel versus standard of care with salvage chemotherapy followed by autologous stem cell transplantation as second-line treatment in patients with relapsed or refractory large B-cell lymphoma (TRANSFORM): results from an interim analysis of an open-label, randomised, phase 3 trial	2022	The Lancet	NCT03575351	Lymphoma	DLBCL

© 2024 Patel K et al. *JAMA Network Open.*

Gene-expression profiling of bortezomib added to standard chemoimmunotherapy for diffuse large B-cell lymphoma (REMoDL-B): an open-label, randomised, phase 3 trial	2019	The Lancet Oncology	NCT01324596	Lymphoma	DLBCL
ROBUST: A Phase III Study of Lenalidomide Plus R-CHOP Versus Placebo Plus R-CHOP in Previously Untreated Patients With ABC-Type Diffuse Large B-Cell Lymphoma	2021	Journal of Clinical Oncology	NCT02285062	Lymphoma	DLBCL
Subcutaneous Rituximab-MiniCHOP Compared With Subcutaneous Rituximab-MiniCHOP Plus Lenalidomide in Diffuse Large B-Cell Lymphoma for Patients Age 80 Years or Older	2021	Journal of Clinical Oncology	NCT02128061	Lymphoma	DLBCL
Rituximab-CHOP With Early Rituximab Intensification for Diffuse Large B-Cell Lymphoma: A Randomized Phase III Trial of the HOVON and the Nordic Lymphoma Group (HOVON-84)	2020	Journal of Clinical Oncology	NA	Lymphoma	DLBCL
Dose-Adjusted EPOCH-R Compared With R-CHOP as Frontline Therapy for Diffuse Large B-Cell Lymphoma: Clinical Outcomes of the Phase III Intergroup Trial Alliance/CALGB 50303	2019	Journal of Clinical Oncology	NCT00118209	Lymphoma	DLBCL
Randomized Phase III Trial of Ibrutinib and Rituximab Plus Cyclophosphamide, Doxorubicin, Vincristine, and Prednisone in Non–Germinal Center B-Cell Diffuse Large B-Cell Lymphoma	2019	Journal of Clinical Oncology	NCT01855750	Lymphoma	DLBCL
Obinutuzumab vs rituximab for advanced DLBCL: a PET-guided and randomized phase 3 study by LYSA	2021	Blood	NCT01659099	Lymphoma	DLBCL
Rituximab plus Lenalidomide in Advanced Untreated Follicular Lymphoma	2018	NEJM	NCT01650701	Lymphoma	Follicular lymphoma
Efficacy, pharmacokinetics, and safety of the biosimilar CT-P10 in comparison with rituximab in patients with previously untreated low-tumour-burden follicular lymphoma: a randomised, double-blind, parallel-group, phase 3 trial	2018	The Lancet Hematology	NCT02260804	Lymphoma	Follicular lymphoma
Response-Adapted Postinduction Strategy in Patients With Advanced-Stage Follicular Lymphoma: The FOLL12 Study	2022	Journal of Clinical Oncology	NCT02063685	Lymphoma	Follicular lymphoma
Randomized Trial of Systemic Therapy After Involved-Field Radiotherapy in Patients With Early-Stage Follicular Lymphoma: TROG 99.03	2018	Journal of Clinical Oncology	NCT00115700	Lymphoma	Follicular lymphoma
Brentuximab Vedotin with Chemotherapy for Stage III or IV Hodgkin’s Lymphoma	2018	NEJM	NCT01712490	Lymphoma	Hodgkin lymphoma
Pembrolizumab versus brentuximab vedotin in relapsed or refractory classical Hodgkin lymphoma (KEYNOTE-204): an interim analysis of a multicentre, randomised, open-label, phase 3 study	2021	The Lancet Oncology	NCT02684292	Lymphoma	Hodgkin lymphoma
PET-adapted treatment for newly diagnosed advanced Hodgkin lymphoma (AHL2011): a randomised, multicentre, non-inferiority, phase 3 study	2019	The Lancet Oncology	NCT01358747	Lymphoma	Hodgkin lymphoma
Ibrutinib plus Bendamustine and Rituximab in Untreated Mantle-Cell Lymphoma	2022	NEJM	NCT01776840	Lymphoma	Mantle cell lymphoma
Lenalidomide maintenance after autologous haematopoietic stem-cell transplantation in mantle cell lymphoma: results of a Fondazione Italiana Linfomi (FIL) multicentre, randomised, phase 3 trial	2021	The Lancet Hematology	NCT02354313	Lymphoma	Mantle cell lymphoma
Positron Emission Tomography–Guided Therapy of Aggressive Non-Hodgkin Lymphomas (PETAL): A Multicenter, Randomized Phase III Trial	2018	Journal of Clinical Oncology	NCT00554164	Lymphoma	Non-Hodgkin lymphoma
Second-Line Tisagenlecleucel or Standard Care in Aggressive B-Cell Lymphoma	2022	NEJM	NCT03570892	Lymphoma	Non-Hodgkin lymphoma (multiple)
Axicabtagene Ciloleucel as Second-Line Therapy for Large B-Cell Lymphoma	2022	NEJM	NCT03391466	Lymphoma	Non-Hodgkin lymphoma (multiple)
Four versus six cycles of CHOP chemotherapy in combination with six applications of rituximab in patients with aggressive B-cell lymphoma with favourable prognosis (FLYER): a randomised, phase 3, non-inferiority trial	2019	The Lancet	NCT00278421	Lymphoma	Non-Hodgkin lymphoma (multiple)

© 2024 Patel K et al. *JAMA Network Open.*

Copanlisib plus rituximab versus placebo plus rituximab in patients with relapsed indolent non-Hodgkin lymphoma (CHRONOS-3): a double-blind, randomised, placebo-controlled, phase 3 trial	2021	The Lancet Oncology	NCT02367040	Lymphoma	Non-Hodgkin lymphoma (multiple)
AUGMENT: A Phase III Study of Lenalidomide Plus Rituximab Versus Placebo Plus Rituximab in Relapsed or Refractory Indolent Lymphoma	2019	Journal of Clinical Oncology	NCT01938001	Lymphoma	Non-Hodgkin lymphoma (multiple)
Rituximab in patients with primary CNS lymphoma (HOVON 105/ALLG NHL 24): a randomised, open-label, phase 3 intergroup study	2019	The Lancet Oncology	NA	Lymphoma	Primary CNS lymphoma
Mogamulizumab versus vorinostat in previously treated cutaneous T-cell lymphoma (MAVORIC): an international, open-label, randomised, controlled phase 3 trial	2018	The Lancet Oncology	NCT01728805	Lymphoma	T-cell lymphoma
Brentuximab vedotin with chemotherapy for CD30-positive peripheral T-cell lymphoma (ECHELON-2): a global, double-blind, randomised, phase 3 trial	2019	The Lancet	NCT01777152	Lymphoma	T-cell lymphoma
Romidepsin Plus CHOP Versus CHOP in Patients With Previously Untreated Peripheral T-Cell Lymphoma: Results of the Ro-CHOP Phase III Study (Conducted by LYSA)	2022	Journal of Clinical Oncology	NCT01796002	Lymphoma	T-cell lymphoma
Randomized Phase III Study of Alisertib or Investigator’s Choice (Selected Single Agent) in Patients With Relapsed or Refractory Peripheral T-Cell Lymphoma	2019	Journal of Clinical Oncology	NCT01482962	Lymphoma	T-cell lymphoma
Addition of four doses of rituximab to standard induction chemotherapy in adult patients with precursor B-cell acute lymphoblastic leukaemia (UKALL14): a phase 3, multicentre, randomised controlled trial	2022	The Lancet Hematology	NCT01085617	Leukemia	Acute lymphoblastic leukemia
Intensified Therapy of Acute Lymphoblastic Leukemia in Adults: Report of the Randomized GRAALL-2005 Clinical Trial	2018	Journal of Clinical Oncology	NCT00327678	Leukemia	Acute lymphoblastic leukemia
Ivosidenib and Azacitidine in IDH1-Mutated Acute Myeloid Leukemia	2022	NEJM	NCT03173248	Leukemia	Acute myeloid leukemia
Oral Azacitidine Maintenance Therapy for Acute Myeloid Leukemia in First Remission	2020	NEJM	NCT01757535	Leukemia	Acute myeloid leukemia
Azacitidine and Venetoclax in Previously Untreated Acute Myeloid Leukemia	2020	NEJM	NCT02993523	Leukemia	Acute myeloid leukemia
Gilteritinib or Chemotherapy for Relapsed or Refractory FLT3-Mutated AML	2019	NEJM	NCT02421939	Leukemia	Acute myeloid leukemia
Sorafenib maintenance in patients with FLT3-ITD acute myeloid leukaemia undergoing allogeneic haematopoietic stem-cell transplantation: an open-label, multicentre, randomised phase 3 trial	2020	The Lancet Oncology	NCT02474290	Leukemia	Acute myeloid leukemia
Quizartinib versus salvage chemotherapy in relapsed or refractory FLT3-ITD acute myeloid leukaemia (QuANTUM-R): a multicentre, randomised, controlled, open-label, phase 3 trial	2019	The Lancet Oncology	NCT02039726	Leukemia	Acute myeloid leukemia
Gemtuzumab Ozogamicin in NPM1-Mutated Acute Myeloid Leukemia: Early Results From the Prospective Randomized AMLSG 09-09 Phase III Study	2020	Journal of Clinical Oncology	NCT00893399	Leukemia	Acute myeloid leukemia
CPX-351 (cytarabine and daunorubicin) Liposome for Injection Versus Conventional Cytarabine Plus Daunorubicin in Older Patients With Newly Diagnosed Secondary Acute Myeloid Leukemia	2018	Journal of Clinical Oncology	NCT01696084	Leukemia	Acute myeloid leukemia
Venetoclax plus LDAC for newly diagnosed AML ineligible for intensive chemotherapy: a phase 3 randomized placebo-controlled trial	2020	Blood	NCT03069352	Leukemia	Acute myeloid leukemia
Azacitidine maintenance after intensive chemotherapy improves DFS in older AML patients	2019	Blood	NA	Leukemia	Acute myeloid leukemia, MDS
Oral arsenic plus retinoic acid versus intravenous arsenic plus retinoic acid for non-high-risk acute promyelocytic leukaemia: a non-inferiority, randomised phase 3 trial	2018	The Lancet Oncology	NA	Leukemia	Acute promyelocytic leukemia
Ibrutinib–Rituximab or Chemoimmunotherapy for Chronic Lymphocytic Leukemia	2019	NEJM	NCT02048813	Leukemia	Chronic lymphocytic leukemia
Venetoclax and Obinutuzumab in Patients with CLL and Coexisting Conditions	2019	NEJM	NCT02242942	Leukemia	Chronic lymphocytic leukemia

© 2024 Patel K et al. *JAMA Network Open.*

Ibrutinib Regimens versus Chemoimmunotherapy in Older Patients with Untreated CLL	2018	NEJM	NCT01886872	Leukemia	Chronic lymphocytic leukemia
Venetoclax–Rituximab in Relapsed or Refractory Chronic Lymphocytic Leukemia	2018	NEJM	NCT02005471	Leukemia	Chronic lymphocytic leukemia
Zanubrutinib versus bendamustine and rituximab in untreated chronic lymphocytic leukaemia and small lymphocytic lymphoma (SEQUOIA): a randomised, controlled, phase 3 trial	2022	The Lancet Oncology	NCT03336333	Leukemia	Chronic lymphocytic leukemia
Ibrutinib plus obinutuzumab versus chlorambucil plus obinutuzumab in first-line treatment of chronic lymphocytic leukaemia (iLLUMINATE): a multicentre, randomised, open-label, phase 3 trial	2019	The Lancet Oncology	NCT02264574	Leukemia	Chronic lymphocytic leukemia
Ublituximab plus ibrutinib versus ibrutinib alone for patients with relapsed or refractory high-risk chronic lymphocytic leukaemia (GENUINE): a phase 3, multicentre, open-label, randomised trial	2021	The Lancet Hematology	NCT02301156	Leukemia	Chronic lymphocytic leukemia
Acalabrutinib Versus Ibrutinib in Previously Treated Chronic Lymphocytic Leukemia: Results of the First Randomized Phase III Trial	2021	Journal of Clinical Oncology	NCT02477696	Leukemia	Chronic lymphocytic leukemia
ASCEND: Phase III, Randomized Trial of Acalabrutinib Versus Idelalisib Plus Rituximab or Bendamustine Plus Rituximab in Relapsed or Refractory Chronic Lymphocytic Leukemia	2020	Journal of Clinical Oncology	NCT02970318	Leukemia	Chronic lymphocytic leukemia
The phase 3 DUO trial: duvelisib vs ofatumumab in relapsed and refractory CLL/SLL	2018	Blood	NCT02004522	Leukemia	Chronic lymphocytic leukemia
Bosutinib Versus Imatinib for Newly Diagnosed Chronic Myeloid Leukemia: Results From the Randomized BFORE Trial	2018	Journal of Clinical Oncology	NCT02130557	Leukemia	Chronic myeloid leukemia
A phase 3, open-label, randomized study of asciminib, a STAMP inhibitor, vs bosutinib in CML after 2 or more prior TKIs	2021	Blood	NCT03106779	Leukemia	Chronic myeloid leukemia
Luspatercept in Patients with Lower-Risk Myelodysplastic Syndromes	2020	NEJM	NCT02631070	Leukemia	MDS
Phase III, Randomized, Placebo-Controlled Trial of CC-486 (Oral Azacitidine) in Patients With Lower-Risk Myelodysplastic Syndromes	2021	Journal of Clinical Oncology	NCT01566695	Leukemia	MDS
Lenalidomide-Epoetin Alfa Versus Lenalidomide Monotherapy in Myelodysplastic Syndromes Refractory to Recombinant Erythropoietin	2021	Journal of Clinical Oncology	NCT00843882	Leukemia	MDS

© 2024 Patel K et al. *JAMA Network Open.*

Supplemental Table 2: Aggregated PROs Used in Reviewed RCTs
Organized by Disease Group

Malignancy (General)	Malignancy (Specific)	General PROs	Disease-Specific PROs	Symptom-Specific PROs
MPN	Essential thrombocythemia	EORTC QLC-C30	MPN-SAF	None
	Polycythemia vera	EORTC QLC-C30	MPN-SAF	None
	Myelofibrosis	EQ-5D, EORTC QLC-C30	MPN-SAF	None
Plasma cell dyscrasia	Light-chain amyloidosis	EQ-5D, EORTC QLQ-C30, SF-36 (v2)	None	None
	Multiple myeloma	EQ-5D, EORTC QLQ-C30, FACT-G, Modified CTSQ, SF-36 (v2)	EORTC QLQ-MY20, FACT-MM, FACT-BMT	FACT-NTX, FACT-F, FACT-P, EORTC-QLQ-CIPN20, BPI-SF, PROMIS-Cancer Fatigue SF
	Smoldering multiple myeloma	FACT-G	FACT-MM	None
	Waldenstrom macroglobulinemia	EQ-5D, EORTC QLQ-C30, FACT-G	None	FACT-An
Lymphoma	DLBCL	EQ-5D, EORTC QLQ-C30, FACT-G	FACT-Lym	FACT-NTX, EORTC-QLQ-CIPN20, QLQ-ELD14
	Follicular lymphoma	EQ-5D, EORTC QLQ-C30	None	None
	Hodgkin lymphoma	EQ-5D, EORTC QLQ-C30, FACT-G	None	FACIT-Dyspnea 10, FACT-NTX
	Mantle cell lymphoma	EQ-5D, EORTC QLQ-C30, FACT-G	FACT-Lym	None
	Non-Hodgkin lymphoma (multiple)	EQ-5D, SF-36 (v2), FACT-G	FACT-Lym, FLymSI-18	None
	Primary CNS lymphoma	EORTC QLQ-C30	EORTC QLQ-BN20	None
	T-cell lymphoma	EQ-5D, EORTC QLQ-C30, FACT-G, TSQM	FACT-Lym	Skindex-29, ItchyQoL, FACT-NTX
Leukemia	Acute lymphoblastic leukemia	GHQ-12	None	None
	Acute myeloid leukemia	EQ-5D, EORTC QLQ-C30, FACT-G	FACT-Leu	FACIT-Fatigue, PROMIS Cancer SF 7a, BFI, FACIT-Dys-SF
	Acute promyelocytic leukemia	FACT-G	None	None
	Chronic lymphocytic leukemia	EQ-5D, EORTC QLQ-C30, FACT-G, MDASI	FACT-Leu, MDASI-CLL, EORTC QLQ-CLL16	FACIT-Fatigue
	Chronic myeloid leukemia	EQ-5D, FACT-G, PGIC, WPAI	FACT-Leu, MDASI-CML	None
	MDS	EQ-5D, EORTC QLQ-C30, FACT-G	None	FACT-An

© 2024 Patel K et al. *JAMA Network Open.*
