# Supplementary material for: Patient-Reported Outcomes in Phase 3 Clinical Trials for Blood Cancers: A Systematic Review
Source: JAMA Netw Open. 2024 Jun 3;7(6):e2414425. doi: 10.1001/jamanetworkopen.2024.14425 (PMC11148691; doi:10.1001/jamanetworkopen.2024.14425)
Supplement: Supplement 2. — Data Sharing Statement [file jamanetwopen-e2414425-s002.pdf]

Data Sharing Statement

Patel. Patient-Reported Outcomes in Phase 3 Clinical Trials for Blood Cancers: A Systematic Review. *JAMA Netw Open*. Published June 03, 2024.
doi:10.1001/jamanetworkopen.2024.14425

Data
Data available: No
